# Nonclinical regulatory immunotoxicity testing of nanomedicinal products: Proposed strategy and possible pitfalls

**DOI:** 10.1002/wnan.1633

**Published:** 2020-04-07

**Authors:** Christina Giannakou, Margriet V. D. Z. Park, Irene E. M. Bosselaers, Wim H. de Jong, Jan Willem van der Laan, Henk van Loveren, Rob J. Vandebriel, Robert E. Geertsma

**Affiliations:** ^1^ Triskelion B.V. Zeist Netherlands; ^2^ RIVM Bilthoven Netherlands; ^3^ CBG‐MEB Utrecht Netherlands; ^4^ Department of Toxicogenomics Maastricht University Maastricht Netherlands

**Keywords:** ICH‐S8, immunotoxicity, in vitro, nanomedicinal product, regulatory

## Abstract

Various nanomedicinal products (NMPs) have been reported to induce an adverse immune response, which may be related to their tendency to accumulate in or target cells of the immune system. Therefore, before their market authorization, NMPs should be thoroughly evaluated for their immunotoxic potential. Nonclinical regulatory immunotoxicity testing of nonbiological medicinal products, including NMPs, is currently performed by following the guideline S8 “Immunotoxicity Studies for Human Pharmaceuticals” of the International Council for Harmonization of Technical Requirements for Pharmaceuticals for Human Use (ICH). However, this guideline does not cover all the immunotoxicity endpoints reported for NMPs in the literature, such as complement activation related pseudo allergy, hypersensitivity and immunosuppression. In addition, ICH‐S8 does not provide any nanospecific testing considerations, which is important given their tendency to interfere with many commonly used toxicity assays. We therefore propose a nonclinical regulatory immunotoxicity assessment strategy, which considers the immunotoxicity endpoints currently missing in the ICH‐S8. We also list the known pitfalls related to the testing of NMPs and how to tackle them. Next to defining the relevant physicochemical and pharmacokinetic properties of the NMP and its intended use, the proposed strategy includes an in vitro assay battery addressing various relevant immunotoxicity endpoints. A weight of evidence evaluation of this information can be used to shape the type and design of further in vivo investigations. The final outcome of the immunotoxicity assessment can be included in the overall risk assessment of the NMP and provide alerts for relevant endpoints to address during clinical investigation.

This article is categorized under:

Toxicology and Regulatory Issues in Nanomedicine > Regulatory and Policy Issues in Nanomedicine
Toxicology and Regulatory Issues in Nanomedicine > Toxicology of Nanomaterials

## Graphical abstract

Proposed strategy for immunotoxicity assessment of nanomedicinal products.

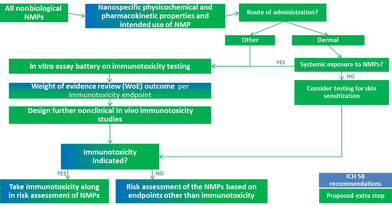

## 1 INTRODUCTION

The number of nanomedicinal products (NMPs) finding their way to the market is steadily increasing (Farjadian et al., 2019; Ragelle, Danhier, Preat, Langer, & Anderson, 2017). In 2015, we reported that 74 NMPs were already on the market while 101 were in the pipeline (Noorlander et al., 2015). NMPs exist in many forms, such as nanocrystals, polymer conjugates, liposomes, and protein nanoparticles. The majority have therapeutic purposes, while a smaller group of mostly metallic NMPs functions as imaging agents. Their common denominator is that they all consist of a 3D nanostructure, less than 1,000 nm across, designed to have specific properties.

Like any other pharmaceutical product, NMPs have to undergo a safety evaluation process before obtaining marketing authorization. Several globally harmonized, regulatory guidance documents are in place to help manufacturers and regulators in the safety assessment process of pharmaceuticals. However, various researchers have expressed that for NMPs, this process may require the development of new or adaptation of existing standards to ensure the quality, safety, and efficacy of such products (Giannakou et al., 2016; Gioria et al., 2018; Halamoda‐Kenzaoui, Holzwarth, Roebben, Bogni, & Bremer‐Hoffmann, 2019). Other regulatory frameworks dealing with nanomaterials such as the EU regulatory framework for chemical substances REACH have also started developing and implementing adapted toxicity testing guidelines for nanomaterials (Rasmussen, Rauscher, Kearns, Gonzalez, & Riego Sintes, 2019). This is due to the now well‐recognized phenomenon that nanomaterials behave differently from conventional chemicals in various test systems (Guadagnini et al., 2015; Kroll, Pillukat, Hahn, & Schnekenburger, 2012).

Upon entering the bloodstream, the surface of NMPs will immediately be covered by biomolecules such as apolipoproteins, sugars, and lipids. The type and amount of biomolecules present on the surface depends on the NMP's physicochemical properties including size, shape, surface charge, surface area‐to‐volume ratio, and surface chemistry (Silva et al., 2017). This pattern of molecules on the surface of NMPs in turn determines how the innate immune cells such as macrophages interact with the NMPs. This is because these cells respond to genetically conserved pathogen‐associated molecular patterns that are recognized by pattern recognition receptors present on the surface of the innate immune cells. Recognition of such patterns results in an immune response, consisting of phagocytosis, an inflammatory response or, eventually, an adaptive immune response.

As a consequence of this interaction, nanomaterials, including NMPs, have the tendency to accumulate in the mononuclear phagocytic system (MPS) including the organs of the immune system (e.g., De Jong et al., 2013; Geraets et al., 2014; Lankveld et al., 2010; Lankveld et al., 2011; Yuan, He, Wu, Fan, & Cao, 2019). In addition, various NMPs have been reported to induce innate or adaptive immunotoxic effects in the literature (e.g., Brand et al., 2017; Moghimi, 2018; Sharma, McLeland, Potter, Stern, & Adiseshaiah, 2018). We therefore concluded earlier that this endpoint requires further attention (Brand et al., 2017).

Regulatory assessment of medicinal products is generally performed according to globally harmonized guidelines defined by the International Council for Harmonization of Technical Requirements for Pharmaceuticals for Human Use (ICH). The guideline that specifically addresses the immune system of nonbiologicals is the ICH‐S8 on immunotoxicity studies for human pharmaceuticals. According to this guideline, initial information on immunotoxicity needs to come from repeated dose in vivo standard toxicity studies (STS), followed by additional dedicated in vivo rodent immunotoxicity studies if needed.

We previously compared the testing recommendations of this guideline to the effects of NMPs reported in the literature (Giannakou et al., 2016). Our conclusion was that immunotoxic effects relevant for NMPs such as complement activation‐related pseudoallergy (CARPA) and hypersensitivity as well as immunosuppression and ‐stimulation indicators such as myelosuppression and inflammasome activation may very well go undetected in the preclinical phase when following the ICH‐S8 guideline. Similar findings were recently reported by Halamoda‐Kenzaoui and Bremer‐Hoffmann (2018), who reviewed the most frequent immune reactions induced by the nanomaterials in vivo and identified the main effects triggered by lipid‐based, polymer‐based and inorganic nanoparticles, as the main categories of nanomaterials used in medicine. For example, they observed that complement activation‐related hypersensitivity reactions and activation of adaptive immune response were the most frequent effects reported for the lipid‐based nanoparticles (e.g., liposomes).

Therefore, we propose a predictive battery of tests for the endpoints relevant for NMPs which are currently not included in the ICH‐S8 guideline, taking into account known pitfalls related to the testing of NMPs. In the following paragraphs, we suggest a number of tests that can be used for this purpose, as well as a strategy on how to integrate these tests in the current ICH‐S8 guideline.

## 2 REGULATORY IMMUNOTOXICITY EVALUATION OF PHARMACEUTICALS

The scope of the ICH‐S8 guideline covers the immunotoxicity evaluation of all nonbiological pharmaceuticals. It should be noted that the immunogenicity of biological NMPs (e.g., monoclonal antibodies) is outside the scope of the ICH‐S8 guideline, as this should be evaluated using the ICH S6 guideline (S6_R1_EMA/CHMP/ICH/731268/1998, June 2011).

**FIGURE 1:**
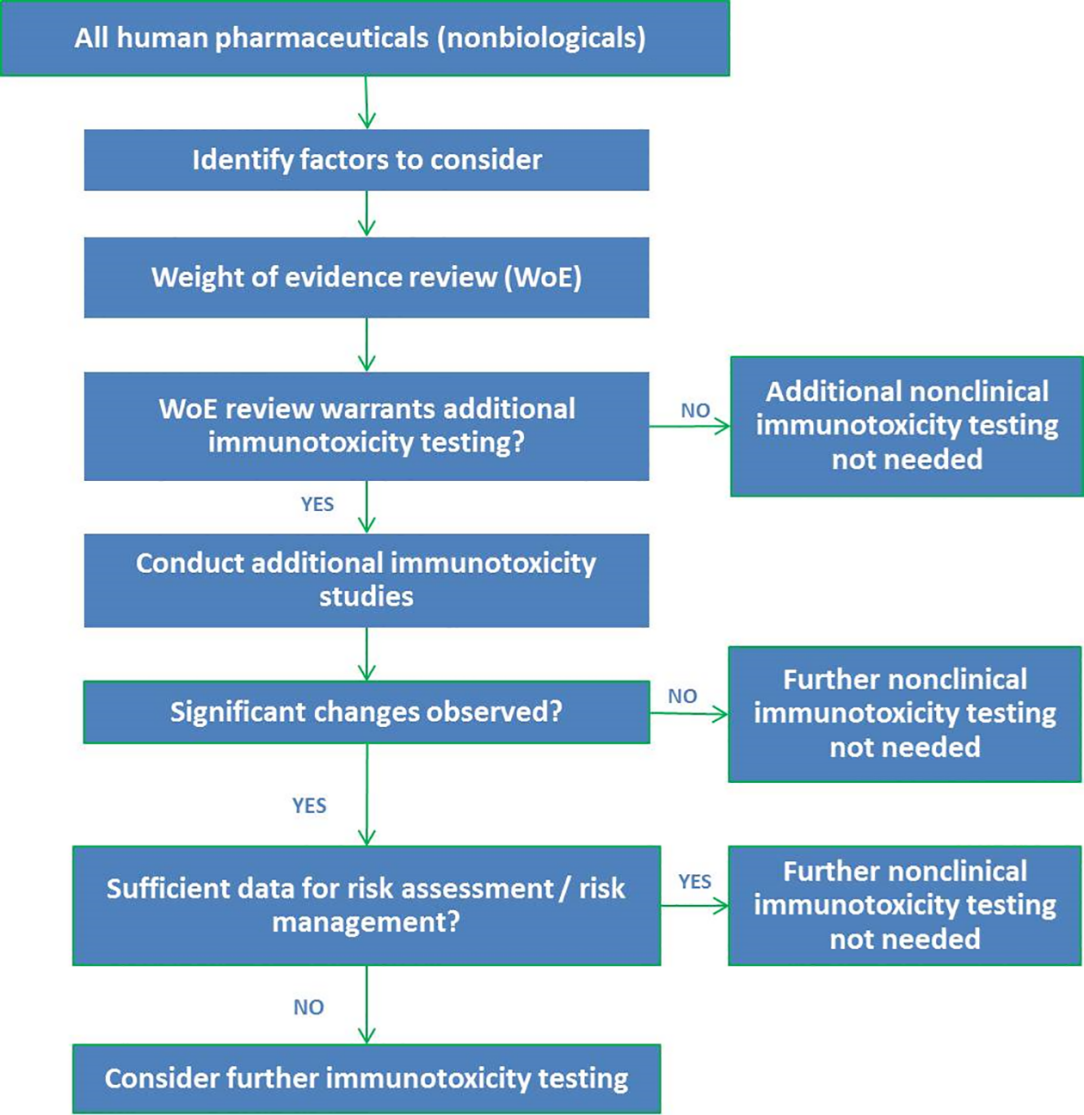
Flow diagram of current regulatory immunotoxicity evaluation of nonbiological pharmaceuticals. Adapted from guideline ICH‐S8

The ICH‐S8 guideline starts with a weight of evidence review of “factors to consider.” These factors consist of (a) findings from STS; (b) the pharmacological properties of the drug; (c) the intended patient population; (d) structural similarities to known immunomodulators; (e) the disposition of the drug; and (f) clinical information (S8_EMA/CHMP/ICH/, n.d.) (Figure 1).

Although the ICH‐S8 guideline was designed primarily for small molecule products, the general flow of the immunotoxicity evaluation appears valid for any pharmaceutical. However, there are a few limitations when the guideline is applied to NMPs. First, the initial weight of evidence review based solely on STS may not provide the necessary alerts for further immunotoxicity testing, particularly for endpoints that have been observed for NMPs, such as CARPA, myelosuppression, inflammasome activation, and hypersensitivity. This is caused by the fact that immunotoxicity related endpoints included in STS are limited to hematology, clinical chemistry, gross pathology, organ weights, and histology. In addition, the ICH‐S8 guideline does not mention specific properties of NMPs that should be taken into account for an adequate immunotoxicity evaluation, such as their tendency to accumulate in the MPS. Furthermore, NMPs have dynamic physicochemical properties that may interfere with various read‐out systems used in toxicity testing, as has also been addressed in various reflection papers on NMPs by the European Medicines Agency (EMA, 2011, 2013a, 2013b).

## 3 PROPOSED REGULATORY IMMUNOTOXICITY EVALUATION OF NMPS

**FIGURE 2:**
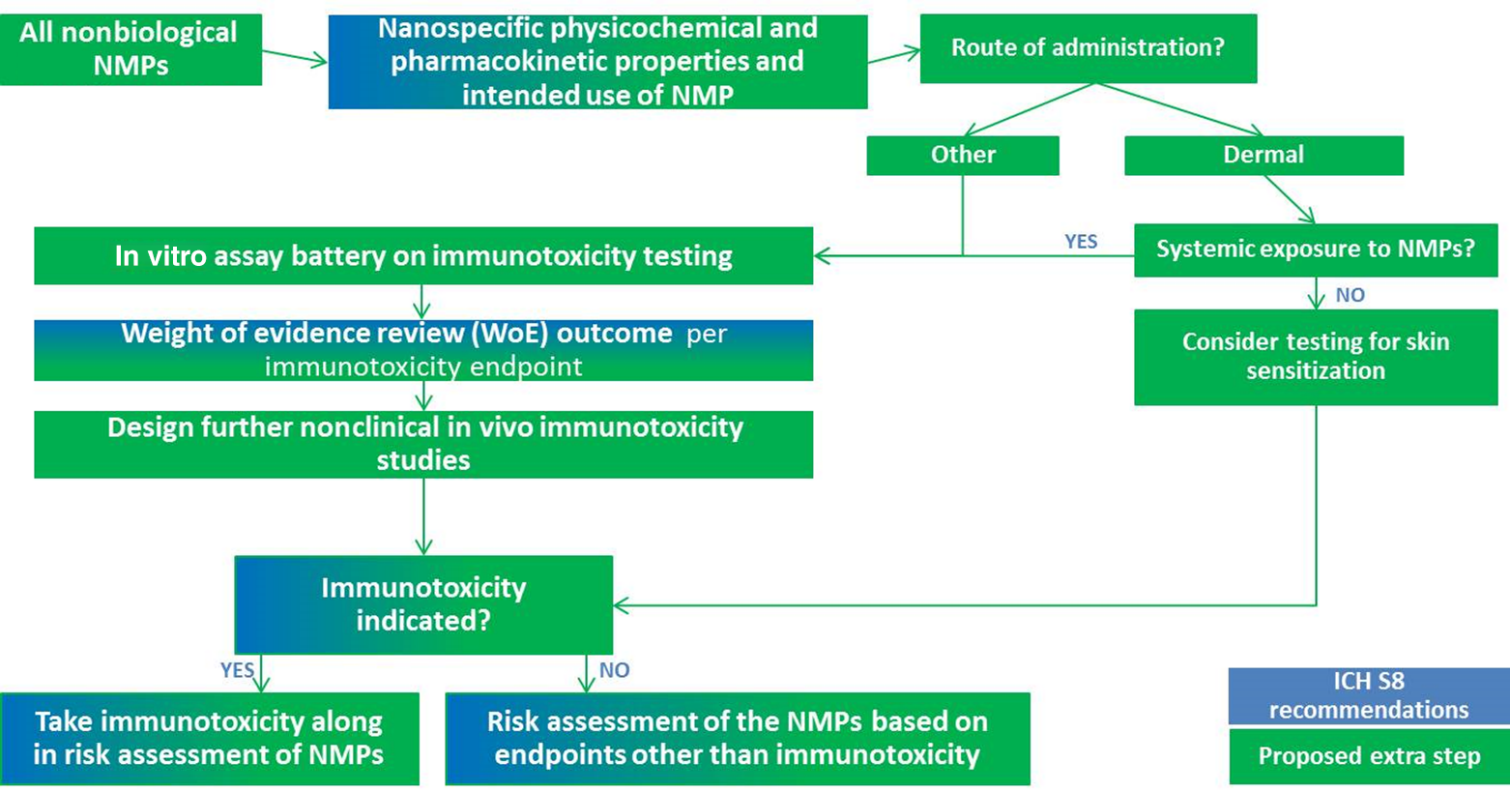
Proposed regulatory immunotoxicity evaluation strategy of nanomedicinal products

To overcome the limitations of the ICH‐S8 for the evaluation of immunotoxicity of NMPs, we propose to adapt the guideline by including additional information in the weight of evidence review. The additional information proposed includes nanospecific physicochemical properties and intended use of the NMPs (e.g., route of administration), determination of the endotoxin content of NMPs as well as information from an in vitro testing battery for CARPA, macrophage function, myelosuppression, lymphocyte function, inflammasome activation, and dendritic cell (DC) antigen presentation (Figure 2).

### 3.1 Scope of the proposed adapted guideline

Due to the complexity and diversity of nanotechnologies applied in medicinal products, to date, there is no formal definition of what is a NMP (Pita, Ehmann, & Papaluca, 2016). In its glossary, EMA describes nanotechnology as “The use of tiny structures less than 1,000 nm across, which are designed to have specific properties” (EMA, 2019). For the strategy as proposed here, we consider nonbiological NMPs only, similar to the current ICH‐S8 guideline. The immunogenicity of biological NMPs (e.g., monoclonal antibodies) should be evaluated using the ICH S6 guideline (S6_R1_EMA/CHMP/ICH/731268/1998, June 2011).

The following paragraphs describe the extra steps of the immunotoxicity evaluation that we propose to include in the ICH‐S8 guideline for the purpose of evaluating nonbiological NMPs.

### 3.2 Nanospecific physicochemical properties

Current regulatory safety evaluation guidelines of pharmaceuticals naturally will already require a thorough description of the chemical structure and pharmacological properties of the active pharmaceutical ingredient (API) and its formulation. However, in the case of NMPs, this information should also include nanospecific information on the characteristics of the NMP as administered, for example, whether the API is administered in nano‐crystal form, or associated with a nano‐carrier or otherwise.

It is well known that biological effects of NMPs (and of nanomaterials in general) are dependent on the physicochemical properties of the particles, such as size (including size distribution) and surface properties. As discussed before, these properties determine the types of biomolecules present on the NMP surface and in turn the pattern recognition by the innate immune cells (Silva et al., 2017). This is a point of attention in the design of generic formulations of existing NMPs. Even a small change in drug formulation may result in different particle properties, a different pattern of biomolecules on the surface and hence, a different immune response. A further complicating factor is that NMP properties tend to be dynamic, that is, they change as they move from one environment to another such as from the drug formulation to the systemic circulation.

Therefore, any study with NMPs, including immunotoxicity studies, needs to carefully characterize the physicochemical properties of the NMP under consideration, preferably throughout the study, and ensure that the results obtained are relevant for the final product as administered to the patient. The six physicochemical properties that were recently reported as most important in modulating the immune response were surface coating, surface chemistry/functionalization, size, chemical structure, surface charge, and hydrophobicity (Halamoda‐Kenzaoui & Bremer‐Hoffmann, 2018). Many of these properties are interrelated; a change in one property will often result in a change in another property. Other particle properties known to modulate toxicity of nanomaterials are shape, physical and chemical stability (including drug release rate from the particle carrier), dissolution rate, and surface reactivity (Halamoda‐Kenzaoui, Baconnier, et al., 2019; Park et al., 2018). The Technical Committee 229 of the International Organization for Standardisation (ISO TC 229) on Nanotechnologies is currently standardizing many of the methods to characterize these properties. Currently, 72 published ISO documents are available while another 40 are under development.

### 3.3 Intended use

Information on the intended clinical administration of the NMP in terms of its route, dose, and frequency, which will be part of any medicine assessment report, can be used to help determine the type of immunotoxicity studies needed and to shape their design.

With regard to the route of administration, the majority of the NMPs in the pipeline or currently on the market are administered intravenously. A smaller proportion is administered by dermal or oral routes (Fornaguera & Garcia‐Celma, 2017). The inhalation route is also being explored for NMPs, for example, antibiotics‐nanomedicine for respiratory tract infections (Ritsema et al., 2018) and nanoparticle‐aided radiotherapy for lung cancer (Ngwa, Kumar, Moreau, Dabney, & Herman, 2017).

In the case of topical application, the type of immunotoxicity studies needed depends on whether the NMP is able to penetrate through the skin or not, that is, whether NMP administration results in systemic exposure or local skin exposure only. This is because immune responses only take place in case there is systemic exposure. For the majority of nanomaterials and NMPs which are not specifically engineered to cross the skin barrier, penetration through the skin is expected to be extremely low, if at all (Jatana & DeLouise, 2014; Palmer & DeLouise, 2016; Schneider, Stracke, Hansen, & Schaefer, 2009; Tak, Pal, Naoghare, Rangasamy, & Song, 2015). Conducting the full range of immunotoxicity studies may not be necessary for NMPs that do not penetrate the skin. However, we do recommend considering the potential of the NMP to induce skin sensitization in case the potential to penetrate the skin is uncertain, for example, because they may be lodged in hair follicles and could eventually penetrate the skin. Various documents have been developed that provide guidance for this specific endpoint (EMA, 2015; ICH, 2015), although their compatibility with NMPs is not specified.

Some NMPs are specifically designed for transdermal drug delivery and have the ability to travel through the different layers of the skin (reviewed by Sala, Diab, Elaissari, & Fessi, 2018). These may become systemically available, for example by uptake in Langerhans cells residing in the skin. If there is systemic exposure, the immunotoxicity evaluation of these products needs to be continued like NMPs administered by any other route resulting in systemic availability. Administration via routes other than skin has been shown to result in systemic exposure for various nanomaterials, although absorbed levels after oral and inhalation administration are generally low (reviewed by ISO, 2019; Kermanizadeh, Balharry, Wallin, Loft, & Moller, 2015).

### 3.4 Endotoxin determination

According to the European Pharmacopoeia and other international medicinal product quality standards, all parenterally administered medicines must comply to certain limits of bacterial endotoxin content. Endotoxins are lipopolysaccharides originating from the outer wall of Gram negative bacteria that can cause activation of the immune system in both humans and experimental animal models. The product limit of endotoxin depends on factors such as the route and dose of administration, patient population, and manufacturing process capability. Most NMPs are administered intravenously, and for these products, the threshold pyrogenic dose of endotoxin per kg body mass is 5 IU (European Pharmacopoeia, 2016).

Since endotoxins can cause activation of the immune system, it is of importance to ensure that the endotoxin content of NMPs is sufficiently low already in the preclinical phase when studying its immunotoxicity in vitro or in vivo. However, the current golden standard for endotoxin determination in medicinal products, the Limulus mebocyte lysate method, is known not to be compatible with all forms of NMP (reviewed by Kettler, Giannakou, de Jong, Hendriks, & Krystek, 2016). We have therefore recently proposed an alternative, analytical method that can determine the presence of endotoxin‐specific fatty acids in NMPs by using ultra high performance liquid chromatography coupled with mass spectrometry (UHP LCMS/MS) (Giannakou et al., 2019). This method has shown sufficient sensitivity and good compatibility with a wide variety of NMPs. It is currently undergoing round robin testing within the EU H2020 research project REFINE.

### 3.5 In vitro immunotoxicity testing

So far, in vitro testing is not part of the ICH‐S8 regulatory guideline for immunotoxicity evaluation of pharmaceuticals. For the purpose of evaluating immunotoxicity of NMPs, we suggest to include into this guideline a battery of in vitro assays to address endpoints of immunotoxicity relevant for NMPs. The outcomes of these assays can be used in the weight of evidence review, together with the information on physicochemical properties and on the intended use of the NMP, to determine whether further in vivo or clinical immunotoxicity testing is needed. The in vitro battery consists of assays for complement activation, macrophage function, inflammasome activation, myelosuppression, lymphocyte function, and DC antigen presentation. We will discuss each of these assays in further detail below, as well as some general considerations on relevant test concentrations and interferences.

#### 3.5.1 Relevant testing concentrations

The concentration range to test immunotoxicity in vitro needs to be pharmacologically relevant, that is, comparable to the exposure at clinical dose levels humans. One way to determine a relevant test concentration range is by using the peak plasma concentration of the product (Cmax) in the clinical setting as a starting point. The testing concentration range then needs to include this Cmax concentration, as well as a few concentrations above the Cmax to account for the potential accumulation of NMPs in the tissues. For example, intravenous administration of iron sucrose to rats resulted in an increase in plasma levels which returned to baseline at 24 hr post administration, while iron levels in spleen and other immune‐related organs continued to increase over the course of 28 days (Elford et al., 2013). This indicates that tissue levels of an NMP can reach much higher concentrations than the Cmax, and these concentrations should be tested for immunotoxicity.

At the same time, the test concentration range needs allow for discrimination between specific immunotoxicity effects and those occurring due to mere cell death. For this reason, cell viability assays should be an integral part of all in vitro immunotoxicity assays. The recommended testing concentrations for immunotoxicity assays are concentrations in the noncytotoxic or subcytotoxic range. We suggest to set the subtoxic concentration for cell viability assays at EC30, that is, the concentration that induces 30% cytotoxicity, in line with what has been proposed for cytotoxicity assays for medical devices by ISO (ISO, 2009). Here, 30% cytotoxicity is used as cut‐off point in view of variation in the nontreated/negative control (100% ± *SD*), that is, an effect on viability >30% is considered a toxic response.

The MTS cell viability assay gives an indication of metabolic activity, while some other cell viability assays measure membrane integrity or other indicators of cell viability. Examples of cell viability assays include alamar Blue, 5 a.m.‐CFDA, WST‐1, Neutral red, and LDH. The most adequate assay depends on the potential of the NMP in question to interfere with the readout system of the assay (see below). It is recommended to use at least two different assays measuring different indicators of cell viability.

#### 3.5.2 Complement activation

**FIGURE 3:**
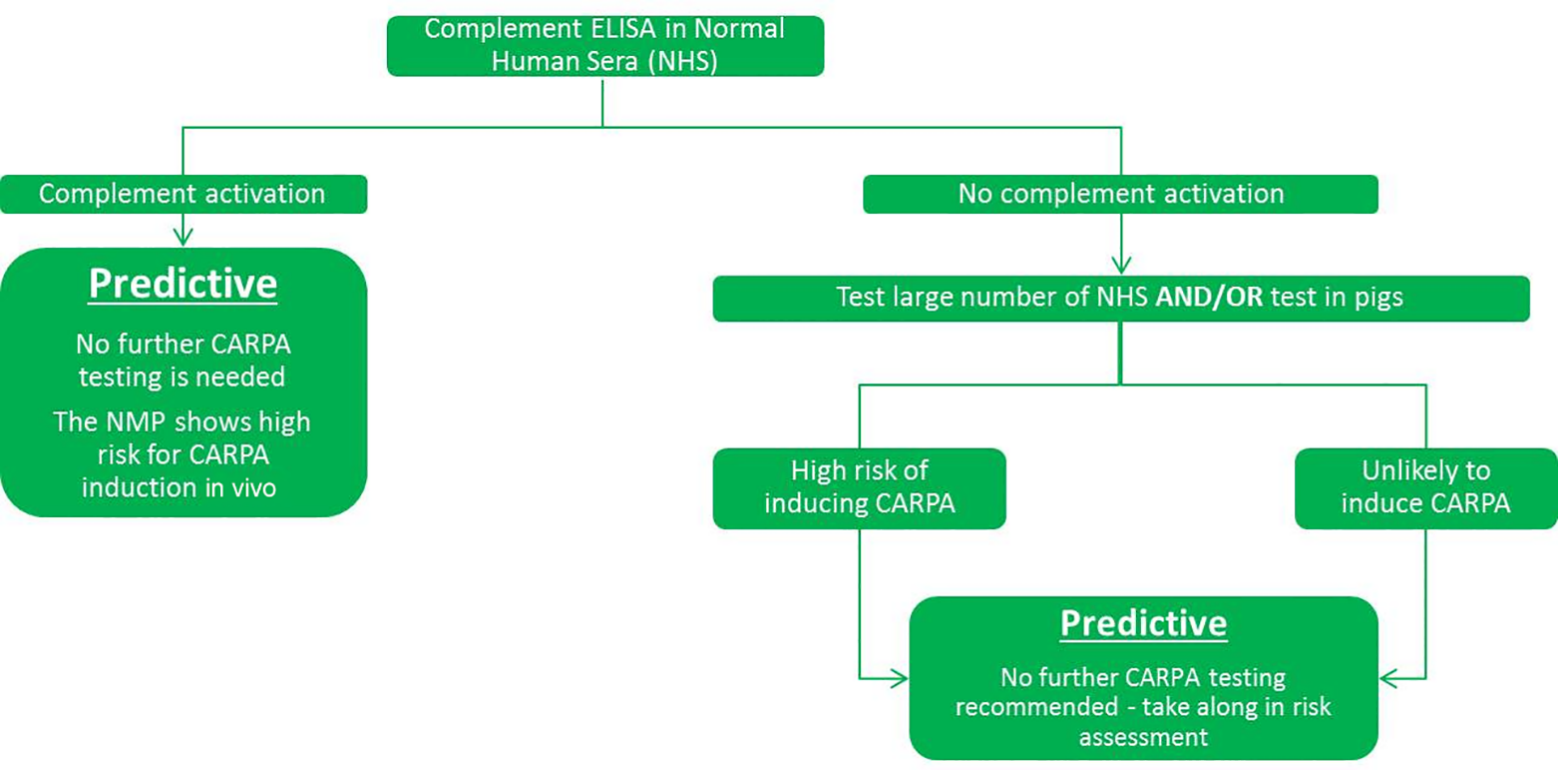
Proposed paradigm for preclinical testing for CARPA induction by nanomedicinal products, modified from the suggested paradigm by Szebeni and Storm (2015)

Complement activation is part of the immune system's response to different triggers, for example, microbial infections, immune complexes, and injured cells. An overblown complement activation response may lead to CARPA. CARPA is a potentially serious adverse event, presented in the clinic as mild to severe allergy‐like symptoms. It has also been reported to occur in patients receiving for the first time treatment with certain types of NMPs (Moghimi, 2018; Szebeni et al., 2012; Szebeni & Moghimi, 2009). It is therefore crucial to test NMPs in the preclinical phase for their ability to induce CARPA in order to be prepared for potential effects in the clinical phase. A paradigm for the nonclinical testing of CARPA has been suggested by Szebeni and Storm (2015) (Figure 3). Several assays are being used today for CARPA determination.

The most popular one is an ELISA‐like assay where components of complement, for example, C3, C5a, C5b, are detected in human serum (Szebeni, 2014). Testing starts using a limited number of sera from single healthy human donors. Based on the outcome of this assay, the need for further testing is determined. If the complement cascade is induced, no further testing is needed. The results are considered predictive and indicate that the NMP under evaluation presents a high risk for CARPA induction in vivo. At this point, the design of the product needs to be reconsidered before continuing the drug development process. On the other hand, if no complement activation is observed during the initial testing, a larger number of normal human sera should be tested. In addition, complement activation should be measured in an in vivo study in pigs and/or dogs (Szebeni, 2014). The outcome of the human sera tests and the in vivo studies can be regarded as predictive for the human situation and no further CARPA testing is needed.

#### 3.5.3 Macrophage function

**FIGURE 4:**
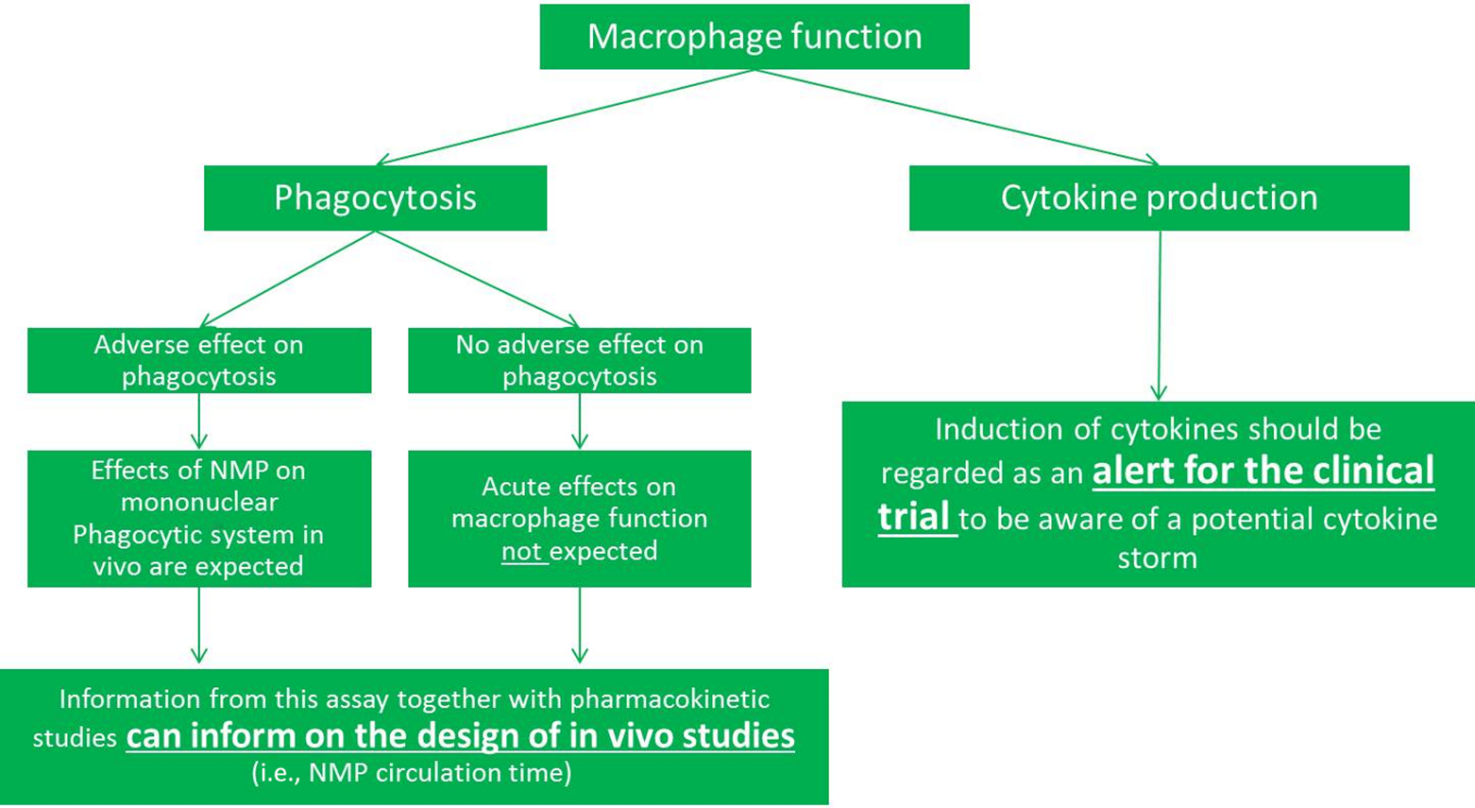
Proposed paradigm for preclinical testing of effects on macrophage function after administration of nanomedicinal products

As discussed before, the first response of the organism to intravenous administered particles is the formation of a protein corona on the particle surface. The protein corona subsequently contributes to the body's recognition and uptake of the particles by phagocytic immune cells such as tissue resident macrophages or monocytes differentiating into macrophages (Epelman, Lavine, & Randolph, 2014; Gustafson, Holt‐Casper, Grainger, & Ghandehari, 2015). Macrophages have been known to uptake foreign materials within a matter of minutes. As a first line of defense, macrophages are of great importance for a well‐functioning immune response (Gustafson et al., 2015). Therefore, to evaluate the safety of NMPs for the immune system, we recommend to include two endpoints of macrophage function: phagocytosis/opsonization capacity and cytokine production (Figure 4).

Significant changes in cytokine production are considered an important alert for immunotoxicity (Dobrovolskaia & McNeil, 2013). Metal oxide nanoparticles that induced an increased production of IL‐1β, IL‐8, and TNFα in normal human peripheral blood mononuclear cells were also reported to lead to congestion in animal spleens and other organs, resembling septic shock in humans. An induction of these cytokines may therefore point to a risk of inducing a systemic inflammatory response or even a cytokine storm during clinical use.

With regard to phagocytosis/opsonization capacity, a positive association was observed between the functioning of the mononuclear phagocyte system in vivo and the clearance of polyethylene glycol (PEG)ylated liposomes (Caron et al., 2013). Across mice, rats, dogs, and humans, the clearance of liposomes was faster with increasing phagocytic capacity, as measured by level of uptake of fluorescent bacteria in whole blood cells. This functioning of the mononuclear phagocyte system can also be studied in vitro, as demonstrated in various monocyte and macrophage studies, which showed a good correlation between the level of phagocytosis and in vivo biodistribution for various nanoparticles (reviewed by Dobrovolskaia & McNeil, 2013).

In phagocytosis experiments in vitro, phagocytic cells such as macrophages are exposed to a test substance and simultaneously or subsequently, their phagocytic capacity is measured by determining the extent of uptake of (fluorescent) bacteria or particles. Performing this assay with MNPs as the test substance serves two purposes:

1. To provide information for the dosing regimen of pharmacokinetic and toxicity studies. For example, a high phagocytic capacity indicates that the MNP will be cleared from the body rapidly and thus a different dose or duration of MNP administration may be needed for in vivo studies.
2. To check for potential adverse effects of an MNP on the phagocytic capacity of the MPS. A low phagocytic capacity may indicate that the MNP impairs the MPS. This in turn may indicate an NMP‐related reduced immune response to pathogens.

#### 3.5.4 Inflammasome activation

Inflammasome activation is one of the initial steps of a specific inflammation response initiated by macrophages and DCs that can follow four distinct modes of action (Guo, Callaway, & Ting, 2015; Sun, Wang, Ji, Li, & Xia, 2013). Dysregulated inflammasome activity via NLRP3, leading to an excessive production of IL‐1β, has been associated with several autoimmune diseases such as inflammatory bowel disease (Villani et al., 2009) and gouty arthritis (So & Busso, 2014). Various nanoparticles, including NMPs, have been shown to specifically induce this NLRP3 inflammasome activation pathway (Sharma et al., 2018).

**FIGURE 5:**
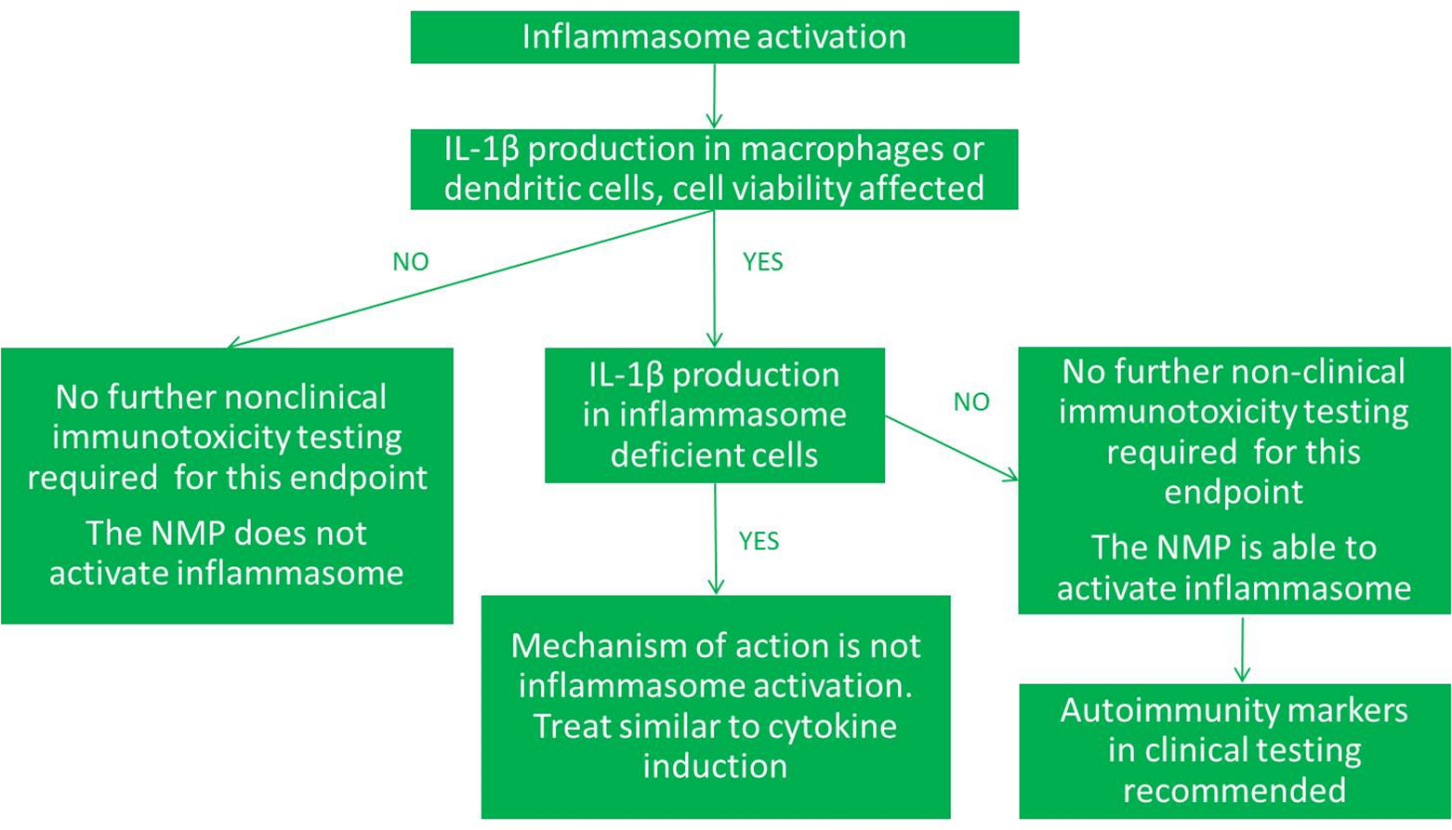
Proposed paradigm for preclinical testing of inflammasome activation after administration of nanomedicinal products

The NLRP3 inflammasome activation pathway can be studied in macrophages and DCs. If exposure does not lead to production of IL‐1β, the NMP is not capable of activating this inflammatory pathway. If exposure to NMPs does lead to an increase of IL‐1β production and concomitant decrease in cell viability, this may indicate a pro‐inflammatory effect of the NMP. To ensure the effect is indeed specifically inflammasome activation, additional testing can be performed in inflammasome deficient cell lines. If no IL‐1β is induced in these inflammasome deficient cell lines, this indicates that the IL‐1β production in the wild type cells was a specific inflammasome activating effect of the NMPs. In that case, it is recommended to include markers of an autoimmune response in further clinical testing of the NMP, such as rheumatoid factor and autoantibodies. On the other hand, if IL‐1β is still induced in the deficient cell lines, this indicates a mechanism other than inflammasome activation was involved, and the results should be interpreted in the same way as effects on any other cytokine (Figure 5).

#### 3.5.5 Myelosuppression

Myelosuppression is an immunotoxicity endpoint entailing a decreased production or function of immune cells by the bone marrow, causing immunosuppression. Myelosuppression is a common dose‐limiting factor of many oncological drugs. These drugs are designed to be cytotoxic and cytostatic and can as such cause myelosuppression upon reaching the bone marrow.

A common assay to check for immunosuppressive potential of oncological drugs is the colony forming unit—granulocyte macrophage assay (Dobrovolskaia & McNeil, 2013). It is an assay commonly used by researchers for screening of myelosuppressive potential of conventional pharmaceuticals and NMPs. In addition, it was assessed as a screening tool of good prediction by the European Committee for Validation of Alternative Test Methods for conventional‐ and nano‐formulations (Dobrovolskaia & McNeil, 2013). In this assay, pluripotent stem cells are isolated from the bone marrow of experimental animals after exposure to the drug, and the growth and differentiation of these cells into macrophages and granulocytes is evaluated. In the ex vivo version of this assay, bone marrow cells are isolated first and are subsequently exposed to the drug, decreasing the number of animals needed. Human derived bone marrow cells have also been used, and have demonstrated to be of good predictive value, also for nanoformulations. The in vitro and ex vivo versions of the assay do not take into account the biodistribution of the medicinal product to the bone marrow and results should therefore be considered in combination with data obtained from pharmacokinetic studies. Another factor to consider is that certain sources of bone marrow cells are more sensitive to immunosuppressive effects than others. For example, use of human and canine cells results in a more predictive outcome than use of rodent cells, and primary cells are more predictive than cancer cell lines (Dobrovolskaia & McNeil, 2013). A lack of effects on myelosuppression can inform on the design of further clinical testing of, for example, oncological drugs, as it possibly allows for the administration of higher doses.

#### 3.5.6 DC function

Maturation of DCs gives information on immunomodulating effects of NMPs on the function of DCs, the bridge between innate and adaptive immunity. Various reports exist on the ability of NMPs to induce maturation of DCs (e.g., Fernandez et al., 2015; Maji et al., 2016; Orlowski et al., 2018), which may in some cases be a beneficial effect. For example, this effect of nanomaterials is investigated for its ability to improve the body's response to vaccines, that is, they function as adjuvants.

Assays for DC maturation inform on the ability of DCs to present antigens and initiate an adaptive immune response. Various sources of DCs can be used for the assay including buffy coats of healthy donor volunteers. Monocytes are purified from these buffy coats, and then differentiated to immature DCs. DCs can also be derived from bone marrow cells isolated from C57BL6 mice (Orlowski et al., 2018). Increased production of IL‐12 and increased expression of surface markers such as CD83 and CD86 indicate an effect of NMPs on maturation on bone marrow cells. In case there is no induction of DC maturation compared to the control, it is considered predictive for the clinical situation. Stimulation of DC maturation in vivo should be considered a strong indication that the NMP is capable of modulating the immune response to antigens. In this case one could consider to investigate the effect of the NMP on the overall immune response in a dedicated in vivo immunotoxicity study such as the T‐cell‐dependent antibody response study. However, it is uncertain whether such a study performed in rodents gives more reliable information on the immunotoxic potential of the NMP in humans than the in vitro results using human cells. Note that a modulation of the immune response to antigens is not necessarily adverse and might actually be a wanted effect when the NMP is used in the context of vaccination.

#### 3.5.7 Lymphocyte function

Lymphocyte function assays can be used to investigate the immunomodulating effect of NMPs on the second line of defense, the adaptive immunity. If exposure to an NMP results in an increase in lymphocyte proliferation response to a mitogen (e.g., LPS or ConA) compared to nonexposed lymphocytes, this may indicate an immunostimulatory effect of the NMP. In contrast, a decreased lymphocyte proliferation response to an antigen may be a sign of immunosuppression, as has been shown for TiO_2_ nanomaterials (Moon et al., 2011) and PEGylated micelle nanocarriers (Dobrovolskaia & McNeil, 2013).

Lymphocyte function can be tested in various ways, including in vitro using lymphocytes purified from buffy coats of healthy donor volunteers (Dobrovolskaia & McNeil, 2013) or ex vivo using splenocytes isolated from mice (Moon et al., 2011). The outcome of this assay will contribute to improving the design of additional nonclinical and clinical studies, for example, adapting the dosing in clinical studies to avoid immunosuppression.

### 3.6 Assay interference

It is well known that nanomaterials and NMPs are able to interfere with different assay read‐outs in different ways, including optical interferences and substrate binding (Guadagnini et al., 2015; Kroll et al., 2012). This interference can limit the use of several assays, especially those relying on optical density or fluorescence measurements. Unfortunately, it is not possible to predict whether a specific NMP is compatible with a specific assay based on its physicochemical properties and this should be assessed on a case by case basis. It is therefore highly recommended in every assay to include controls for possible interference of the NMP with the reagent and for interference with the developed color/fluorescent product. One way to test for interference of the NMP with the assay reagent or reaction product is to determine the absorbance or fluorescence of the NMPs alone in testing conditions with the reagent or reaction product, but without cells. In case the interference of the NMPs with the readout is high (e.g., >10 or 20% absorption by the NMP alone), it does not allow for trustworthy results. In these cases, alternative assays, if available, should be used for the determination of the specific parameter. In cases where the level of interference is low, results may still be used by correcting for these levels of interference.

### 3.7 Weight of evidence review and further development

Review of results from the in vitro studies above in combination with the outcome of the STS, along with NMP characterization and pharmacokinetic data, will already give a good indication on whether adverse immune effects of the NMP can be expected and if so, on what type of adverse immune response. This should help investigators and risk assessors to determine their further course of action, that is, whether to treat any observed effects as alerts for clinical trials, execute specialized in vivo functional immunotoxicity studies such as a T‐cell dependent antibody response study, or reconsider further development of the NMP altogether. Naturally, during regulatory risk assessment of the NMP, the information on the immune effects should be considered together with information on other toxicity endpoints.

## 4 CONCLUSIONS AND FUTURE PERSPECTIVE

Endpoints of immunotoxicity relevant for NMPs that are not included in the ICH‐S8 guidance for immunotoxicity assessment of pharmaceuticals can be appropriately addressed with a set of in vitro assays with fair or good predictive value for humans in addition to the results of the STS.

First, before testing NMPs in vitro, we recommend to characterize a minimum set of physicochemical properties most important in modulating the immune response: surface coating, surface chemistry/functionalization, size, chemical structure, surface charge, and hydrophobicity.

Second, we propose an integrated testing strategy outlining which in vitro assays can be used and how their results inform on the expected adverse immune response of the NMP under consideration. To this end, we have selected in vitro assays which we believe are most fit for their purpose, that is, assays that have been demonstrated to offer a fair or good prediction of an adverse immune response and which are in a relatively far advanced stage of standardization. It needs to be noted that not all effects observed in these assays should be considered adverse. For example, not every increase in cytokines observed in macrophages exposed to NMPs implies that an immunotoxic response to the product will occur. A small increase may simply be needed for the clearance of the product. An exact cut‐off point between adverse and nonadverse effects cannot be given at this point in time. The outcome of an assay like cytokine induction therefore only serves as an alert rather than providing a definite answer on whether the NMP will cause adverse effects. It may be possible in future to determine a cut‐off point between adverse and nonadverse effects if a large amount of comparable data becomes available from using standardized testing methods. A number of steps need to be taken before the proposed strategy is fit for regulatory use. Even more than half a century after the introduction of the 3Rs—reduction, refinement, and replacement—with regard to the use of animal studies, the safety assessment of pharmaceutical products still heavily relies on in vivo data. Also for immunotoxicity, current guidelines on the evaluation of pharmaceuticals, published in 2005, recommend in vivo testing (ICH, 2006). However, since the publication of these guidelines, research and technology have developed to a more advanced level and in vitro techniques are now slowly replacing or supplementing in vivo studies in regulatory safety assessments. For example, for the safety evaluation of chemical substances, OECD guidelines have been adopted for nonanimal models of skin corrosivity (OECD 430,431,435), skin irritation (OECD 439), severe eye irritation (OECD TG 437, 438,460, 491, 492), phototoxicity (OECD 432), in chemico and in vitro skin sensitization (OECD 442C, D, E), mutagenicity (OECD 471, 480, 481, 476, 490, 473, 487, 482, 479), dermal absorption (OECD 428) and developmental and reproductive toxicity (OECD 455, 457, 493, 458, 456). Several of these documents are already adopted by the European Chemicals Agency, although not all can be used as a stand‐alone assay to address these toxicity endpoints. The European Directorate for the Quality of Medicines and Health Care is also moving more toward the use of 3Rs in several of its activities, including the elaboration of the European Pharmacopoeia, the Biological Standardisation Programme and the Official Medicines Control Laboratory network. It appears that the global movement toward more predictive safety testing without the use of animal models is inevitable. In response to this, the EMA published a guideline on the principles of regulatory acceptance of 3Rs testing approaches (EMA, 2016). These principles include a correct measure or prediction of the biological effect of interest (relevance), availability of a standard protocol, robustness of the test method, and others.

The in vitro assays of immune related toxicity endpoints proposed in our strategy are considered to provide a good or fair prediction of the in vivo situation (Dobrovolskaia & McNeil, 2013; Szebeni, 2014), or at least allow for the detection of known mechanisms of immunotoxicity. Many of the assays have already been tested with several different NMPs. Nevertheless, for regulatory purposes, these assays need to be robust and well‐characterized with regard to their applicability domain, and would therefore benefit from further standardization. The Horizon2020 project REFINE is further developing the method for determination of LPS using LCMS/MS, and the assays for inflammasome activation, DC maturation and cytokine production by creating SOPs and performing round robin studies involving multiple laboratories.

The strategy as proposed reflects the current state of the art of knowledge on the relation between NMP exposure and immunotoxic responses and the assays available to address these immunotoxicity endpoints. To the best of our knowledge, this is the first time a strategy is proposed for the immunotoxicity evaluation of NMPs. More complex assays such as precision‐cut organ slices and co‐cultures of different types of cells exist that address the same immunotoxicity endpoints, but experience with these systems is too limited to conclude on their superior value compared to the in vitro assays suggested.

The strategy does not encompass all endpoints of immunotoxicity. For example, for auto‐immunity, knowledge on the biology is lacking or in its infancy, even for conventional chemicals or pharmaceuticals. It has been suggested that inflammasome activation plays a role in certain auto‐immune diseases (Mangan et al., 2018), but more research is needed to confirm this link.

We propose to use the strategy outlined above as an Integrated Approach to Testing and Assessment (IATA) (OECD, 2016; Sakuratani, Horie, & Leinala, 2018). In practice, this means that when additional relevant information, such as in chemico or in silico data becomes available, this can be included in the risk assessment to substantiate the conclusion on potential immunotoxicity. In that case, it is important to develop a fixed data interpretation procedure (DIP) in order to harmonize the analysis of results from a wide range of sources (see Box 1).

A concept popular within safety assessment of chemicals and strongly supported by ECHA in the frame of REACH is read‐across, where safety data of one chemical can be extrapolated to a group of structurally similar chemicals. The current in vitro immunotoxicity testing strategy may be of use for applying a similar concept to NMPs—using in vitro results of the strategy to support read‐across data from one NMP for which sufficient in vivo or clinical immunotoxicity data is available to another NMP for which this data is lacking. Read‐across is currently a popular topic of investigation for nanomaterials (Arts et al., 2015; Oomen et al., 2015; Park et al., 2018). Nevertheless, guidance on how to apply the concept to nanomaterials in a regulatory context is still a matter of debate and the main objective of the ongoing EU H2020 project GRACIOUS. Read‐across of immunotoxicity data for NMPs will only be possible when relations between NMP physicochemical characteristics and immunotoxicity are clear. This requires testing of series of NMPs, with systematic variations in size, charge, and so forth.

BOX 1 **INTEGRATED APPROACHES TO TESTING AND ASSESSMENT AND DATA INTERPRETATION PROCEDURE**

Integrated testing approaches similar to the proposed immunotoxicity testing strategy have been part of regulatory safety assessment of conventional chemicals for several years already (Hartung, Luechtefeld, Maertens, & Kleensang, 2013; Rovida et al., 2015; Sakuratani et al., 2018; Worth & Patlewicz, 2016). These integrated approaches to testing and assessment (IATA) are especially popular where regulatory safety assessments depend on nonanimal data, such as the safety assessment and of cosmetic ingredients under the Cosmetics directive (European Commission, 2017; Jatana & DeLouise, 2014; Jaworska, 2016; OECD, 2016). The OECD describes IATA's as “pragmatic, science‐based approaches for chemical hazard characterisation that rely on an integrated analysis of existing information coupled with the generation of new information using testing strategies” (http://www.oecd.org/chemicalsafety/risk‐assessment/iata‐integrated‐approaches‐to‐testing‐and‐assessment.htm). This type of assessment approach always involves a certain level of expert judgment, but some elements can be standardized, such as rules for data interpretation. In 2016, OECD published a guidance document on the use of IATA's for testing and assessment of chemical substances (OECD, 2016; Sakuratani et al., 2018), which includes an explanation of the concept of fixed data interpretation procedures. An example of an IATA approach with a fixed data interpretation procedure already incorporated in regulatory guidelines is the skin sensitization IATA (OECD, 2016).

Another concept that is gaining great interest in the world of nanomaterials and NMPs is that of safer‐by‐design (Accomasso, Cristallini, & Giachino, 2018). This concept stimulates the consideration of safety aspects throughout the development process, already from an early development stage, providing opportunities to prevent safety issues at later stages of development. Here, the proposed strategy may be of great value. Rather than performing the suggested assays only at a late stage of development for regulatory purposes, these assays could provide useful information on the potential adverse immune effects of NMPs at an early stage, helping to shape decisions on the further development of NMPs in the pipeline.

## CONFLICT OF INTEREST

The authors have declared no conflicts of interest for this article.

## AUTHOR CONTRIBUTIONS

**Christina Giannakou:** Conceptualization; investigation; methodology; writing‐original draft. **Margriet Park:** Conceptualization; funding acquisition; investigation; methodology; resources; supervision; writing‐original draft; writing‐review and editing. **Irene Bosselaers:** Conceptualization; methodology; writing‐review and editing. **Wim deJong:** Conceptualization; funding acquisition; investigation; methodology; supervision; writing‐review and editing. **Jan Willem van der Laan:** Conceptualization; methodology; writing‐review and editing. **Henk van Loveren:** Conceptualization; funding acquisition; methodology; supervision; writing‐original draft; writing‐review and editing. **Rob Vandebriel:** Investigation; methodology; supervision; writing‐review and editing. **Robert Geertsma:** Conceptualization; funding acquisition; resources; supervision; writing‐original draft; writing‐review and editing.

## RELATED WIREs ARTICLES

In vitro to in vivo benchmark dose comparisons to inform risk assessment of quantum dot nanomaterials

Mapping of the available standards against the regulatory needs for nanomedicines

Overview of the blood compatibility of nanomedicines: A trend analysis of in vitro and in vivo studies

National Cancer Institute Alliance for nanotechnology in cancer‐Catalyzing research and translation toward novel cancer diagnostics and therapeutics
